# Effect of a sustained-release formulation of β-alanine on laboratory parameters and paresthesia in recreational trained men: a randomized double-blind placebo-controlled study

**DOI:** 10.3389/fnut.2023.1213105

**Published:** 2023-09-12

**Authors:** Ana Belén Maestre-Hernández, Silvia Pérez-Piñero, Francisco Javier López-Román, Luis Andreu-Caravaca, Antonio J. Luque-Rubia, Domingo J. Ramos-Campo, María José Díaz-Silvestre, Vicente Ávila-Gandía

**Affiliations:** ^1^Sports Physiology Department, Faculty of Health Sciences, UCAM Universidad Católica San Antonio de Murcia, Guadalupe, Spain; ^2^Primary Care Research Group, Biomedical Research Institute of Murcia (IMIB-Arrixaca), Murcia, Spain; ^3^Facultad de Deporte. UCAM, Universidad Católica de Murcia, Guadalupe, Spain; ^4^LFE Research Group, Department of Health and Human Performance, Faculty of Physical Activity and Sport Science-INEF, Madrid, Spain

**Keywords:** β-Alanine, laboratory parameters, recreational athletes, paresthesia, tolerability

## Abstract

**Introduction:**

Beta-alanine is a non-essential amino acid that has been a focus of increasing research by its role as ergogenic aid to improve muscle performance.

**Methods:**

A randomized, double-blind and controlled trial was conducted to determine the effect of a nutritional supplement of a sustained-release formulation of β-alanine in recreational trained men. The active product was an innovative sustained-release β-alanine microgranules powder blend, administered at high doses (15 g/day) divided into 3 intakes during 30 days. There were 10 participants in the experimental group and 9 in the placebo group, with a mean age of 22.5 ± 3.3 years. Participants were testing at baseline and at the end of study.

**Results:**

In the β-alanine group, there were statistically increases in serum triglycerides, LDL-cholesterol, and urea nitrogen at the end of the study as compared with baseline, although there were no differences with the control group. The occurrence of paresthesia, described above all as tickling, was the majority but presented VAS score less than 3/10 in almost all subjects.

**Discussion:**

More studies are required to evaluate the changes in blood parameters that can be caused by high intake of β-alanine during a long period of time.

**Clinical trial registration:**

ClinicalTrials.gov, identifier (NCT05334121).

## 1. Introduction

Beta-alanine is a non-essential amino acid that has been a focus of increasing research for its combination with L-histidine to form the dipeptide carnosine, which is characterized by its role as ergogenic aid to improve muscle performance (1). Carnosine synthase synthesizes carnosine from β-alanine and L-histidine, and carnosinase is involved in the degradation of carnosine. Carnosine exerts an ergogenic effect leading to an improvement of muscular performance in high-intensity exercise in both trained and untrained subjects (1). Involvement of carnosine in different physiological functions in the homeostasis of contracting muscle cells has been demonstrated, especially during high rates of anaerobic energy delivery (1). A number of actions are related with muscle carnosine as antioxidant and antiglycation agent, increasing sensitivity of muscle contraction to calcium and enhancing the capacity of intracellular H+ buffering, but especially carnosine is involved in attenuation of acidosis (2, 3).

Systematic reviews and meta-analyses have been conducted to assess capacity and exercise performance associated with supplementation with β-alanine, all of which have demonstrated a significant overall beneficial effect but influenced by different modifying variables related to the type of exercise, training status, exercise duration, intermittent or continuous exercise, and co-supplementation with other products (e.g., sodium bicarbonate, carbohydrates) (4–6).

The use of β-alanine supplementation focused on increasing the intramuscular content of carnosine to optimize its potential ergogenic effects has attracted strong interest in recent years (7). The maximum capacity of muscle to store carnosine remains unknown (8), which means that total performance gains by β-alanine supplementation can be greater than what we currently know. Chronic supplementation is conditioned by paresthesia, a harmless sensory side effect of itching (9–11) that limits the maximum single dose tolerable (12) and therefore the maximum daily dose feasible, consequently pro-longing treatment time.

Paresthesia (uncomfortable tingling sensation) is a non-harmful side effect associated with β-alanine supplementation. The exact underlying molecular mechanisms of paresthesia remain elusive, although activation of strychnine-sensitive glycine receptor sites associated with glutamate sensitive N-methyl-D-aspartate receptors in the central nervous system (CNS) (10, 13) has been proposed. In an attempt to prevent paresthesia, sustained-release or controlled formulations have been introduced, given the close relationship between paresthesia and time-to-peak blood concentration of β-alanine. Sustained-release formulations may have the advantage of tolerability of single higher doses of β-alanine, which may result in allowing the use of larger daily doses. In a study in healthy volunteers, de Salazar et al. (14) reported that 8 g in a single oral dose of an innovative sustained-release β-alanine formulation (BETAFOR3MAX®) provided more efficient release kinetics with a 2-fold increase in bioavailability compared to the group that did not take the product without a significant increase in paresthesia. This dose has been the highest acute dose reported in a single administration, higher than the standard recommended daily dose of 6.4 g. In another study of Ávila-Gandía et al. (15), the administration of 5 g of this same formulation, four times daily during 1 week to world tour cyclists during overreaching training, helped to attenuate 10-min time trial performance losses with no side effects or paresthesia.

Moreover, there are other variables that may exhibit changes after the ingestion of a high dose of β-alanine. Among them, body composition is included, which authors such as Kern et al. (16) and Walter et al. (17) have observed changes following the intake of this substance. Thus, it is necessary to control this variable to quantify its potential alterations.

In the present study, a high dose of β-alanine (15 g/day) in a sustained-released formulation was administered to recreational trained men over a period of 30 days. We evaluated the potential effects of this formulation on laboratory parameters, paresthesia and anthropometric variables. To our knowledge, the impact of a daily dose of 15 g of β-alanine in a sustained-released formulation given for a month in a population of non-professional athletes has not been tested before.

## 2. Methods

### 2.1. Design of the study and participants

The study was designed as a randomized, blinded, controlled trial with two parallel groups, which was carried out at the Department of Sports Physiology of the Faculty of Medicine of San Antonio Catholic University of Murcia (UCAM), in Murcia (Spain). The primary objective of the study was to determine whether the administration of a dietary supplementation with a sustained-release formulation of β-alanine, 15 g/day during 30 consecutive days to recreational trained men was associated with changes in complete blood count, lipid profile, liver and renal function tests, the occurrence of paresthesia, as well as in anthropometric variables as compared with placebo.

Participants in the study were recreational subjects who had been volunteering for other studies previously conducted at the UCAM and registered in the institutional database. Inclusion criteria were: healthy adult men, aged 18–40 years old, being recreational trained men with regular three training sessions per week, and at least one year of continuous training. All participants were of Caucasian ethnicity. Exclusion criteria were: subjects with chronic diseases, severe disorders or terminal illness, those suffering from any type of injury that prevented them from regular training in the month prior to the study, previous consumption of β-alanine supplements, and failure to fully understand the study and to provide informed consent.

The Research Ethics Committee of UCAM approved the study protocol (registration code CE032003, date of approval February 24, 2017). The study was registered in the ClinicalTrials.gov (NCT05334121). All participants signed the written informed consent form.

### 2.2. Intervention and study procedures

Participants were assigned at random to oral supplementation with a sustained-release formulation of β-alanine (experimental group) or supplementation with placebo (control group). Randomization was performed by an independent researcher using a computer-generated randomization list generated by *Epidat 3.1 software*.

Participants randomized to the active supplement group received the active product (BETAFOR3MAX®, Martínez Nieto, S.A., Cartagena, Spain), which is a sustained-release β-alanine microgranules powder blend that should be consumed in three daily intakes of 5 g each, just before breakfast, lunch, and dinner (total 15 g of β-alanine per day) during a 30-day period. Two thirds of the content of the granule was β-alanine and the remaining one third was coated for sustained release. The technology of the sustained-release system has been previously described (14). Participants assigned to the control group received an identically appearing microgranular powder based on uncooked wheat semolina from *Triticum durum*, and followed the same regimen. Participants were provided a container with the microgranular powder of the study product and a dispenser, and were instructed to dissolve one scoop (5 g β-alanine or placebo) in 150 mL of water for oral intake. The same company manufactured the study products, which were presented in opaque sealed containers, labeled with the code of the study and the number of the participant. Containers of the study products were weighed for the assessment of compliance. In relation to compliance, at least 80% of the total 90 servings should have been consumed, with a maximum of 18 missed servings. The amount remaining in grams at the end of the study was subtracted from the total amount for the assessment of compliance.

Participants visited at baseline before administration of the study products (visit 1) and after 30 days (final visit, visit 2). During the 30-day study period, they were instructed to the need to maintain the dietary habits and to be compliant with their scheduled training exercise practices three times a week. At the baseline visit, fulfillment of the inclusion criteria was confirmed, participants were requested to sign the informed consent, and the study product was provided. Variables included laboratory tests, assessment of paresthesia and anthropometric data, which were evaluated at visits 1 and 2.

At each study visit, blood samples (10 mL) from the antecubital vein were drawn in fasting conditions (at least 12 h) for laboratory testing. It was recommended not to perform severe or moderate physical exercise at least during 1 day prior to laboratory analysis, and current smokers to refrain from smoking for at least 1 h before analysis. Collection of blood samples included the use of tripotassium EDTA tubes, centrifugation at 4500 rpm for 5 min at 4°C, separation of the supernatant, which was kept frozen (−20°C) until analysis. Beta-alanine was measured using LC–MS/MS liquid chromatography–tandem mass spectrometry (Clinical Analysis Laboratory, Centro medico Virgen de la Caridad, Cartagena, Spain) and results expressed as μmol/L. An automated hematology analyzer (System XS-1000i, Sysmex Corporation, Kobe, Kansai, Japan) was used for blood cell count. Biochemical analyses, including lipid profile [total cholesterol, triglycerides, high-density lipoprotein (HDL) cholesterol, and low-density lipoprotein (LDL) cholesterol]; liver function tests [alanine aminotransferase (AST), aspartate aminotransferase (AST), and gammaglutamyl transpeptidase (GGT); and renal function tests] (serum creatinine, urea nitrogen, and uric acid) were determined using a standard clinical chemistry analyzer (BA 400®, BioSystems, Barcelona, Spain).

Evaluation of paresthesia was performed immediately after each blood sample collection (at visits 1 and 2) using a questionnaire in which the intensity and qualitative description of subjective symptoms of paresthesia were recorded. A visual analog scale (VAS) was used to assess the intensity of paresthesia. The scale was a standard 10-cm line, with vertical marks at each ending, one representing “the most intense sensation imaginable” and the other “no unusual sensation.” The VAS score was calculated as the segment length from the distance at which the subject made a mark (his/her perception of intensity) to the low end. Qualitative description of paresthesia included the selection of six possible sensations, including pins-and-needles, pain, numbness, itching, shiver, and tickling. There was also an empty space in the questionnaire, so that participants were able to describe other symptoms. Moreover, the opinion of participants regarding the product that they had received during the study was requested.

Anthropometric variables included height, weight, body mass index (BMI) and fat, lean, and muscle mass. Height was measured using a wall mounted stadiometer, and body weight using a calibrated Salter weighing scale. Three measurements were taken and the average was calculated. Foot-to-foot bioimpedance analysis (BIA) was used to determine fat mass, lean mass, and muscle mass using Tanita BC-420MA BIA analyzer (Tanita Corporation, Arlington Heights, IL, United States). The conditions considered for the BIA measurement were: (1) Overnight fasting or avoidance of food and/or beverages during the 4 to 8 h prior to measurement, (2) Urinate before measurement, (3) No exercise (24 h prior), (4) No alcohol or diuretics (48 h prior), (5) No menstruation, (6) No metal objects and/or prostheses, (7) Minimal clothing should be worn, (8) The electrode-skin contact area must be clean, (9) Perform the measurement after 5 min of rest of the subject, and (10) Take the measurement at the same time as the previous measurement. Compliance with the study product as well as adverse events were evaluated at visit. To check compliance with the intake, participants were required to return the excess product and it was counted to see if there was adequate consumption of the product. It was established that the actual consumption should be above 90% of the theoretical consumption.

### 2.3. Statistical analysis

The sample size was calculated according to the difference in VAS score of paresthesia as the main variable of the study. Considering a standard deviation of this variable of 2.32 μmol/L reported in a similar population de Salazar et al. (14), for a precision of 2.75 with an alpha risk of 5% and statistical power of 80%, 9 subjects in each group. If we consider a 10% loss to follow-up, 10 subjects will be needed in each group. A convenience sample of 24 participants was chosen. A per-protocol (PP) analysis was performed. The PP population included all eligible subjects who completed the 30-day study period. Frequencies and percentages were used to express categorical variables and mean ± standard deviation (SD) for continuous data. The distribution of categorical variables in the two study groups was compared with the chi-square test or the Fisher’s exact test, and the distribution of quantitative variables at baseline with the *Student’s t test*. Repeated measures ANOVA for paired samples was applied to assess changes of variables during the study period, including within-subject factor (data at baseline and at 30 days) and between-subject factor (supplementation with the active product and supplementation with placebo), considering age as a covariate. The Tukey’s procedure or Bonferroni’s correction were used in the post-hoc analyses. A *p* < 0.05 value was accepted as statistically significant. Deviations from the range of normal values of laboratory parameters were analyzed. Data analysis was carried out using SPSS version 25.0 (IBM Corp., Armonk, NY, United States).

## 3. Results

### 3.1. Anthropometric characteristics

As shown in Table 1, within-group and between-group (time x group interactions) differences in weight, body mass index (BMI), fat mas, lean mass, and muscle mass between the study groups were not found.

**Table 1:** Number of subjects presenting paresthesia and VAS score of the intensity of paresthesia in the study sample.

		*N* (%)	VAS score Mean ± SD	Baseline differences *p* value	Between-group differences Time x Product *p* value
Experimental group	Visit 1 (baseline)	1 (10)	0.10 ± 0.03	0.001	0.006
	Visit 2 (end of study)	9 (90)	1.86 ± 1.74		
Control group	Visit 1 (baseline)	1 (11.1)	0.56 ± 0.17	1.00	
	Visit 2 (end of study)	1 (11.1)	0.56 ± 0.17		

N: number of subjects. Data as mean and standard deviation (SD).

### 3.2. Compliance and adverse events

Compliance above 80% of the study product was confirmed in all participants. The study products were well tolerated and except for subjective symptoms of paresthesia, other adverse events were not registered.

### 3.3. Disposition of study participants

**Figure 1:**
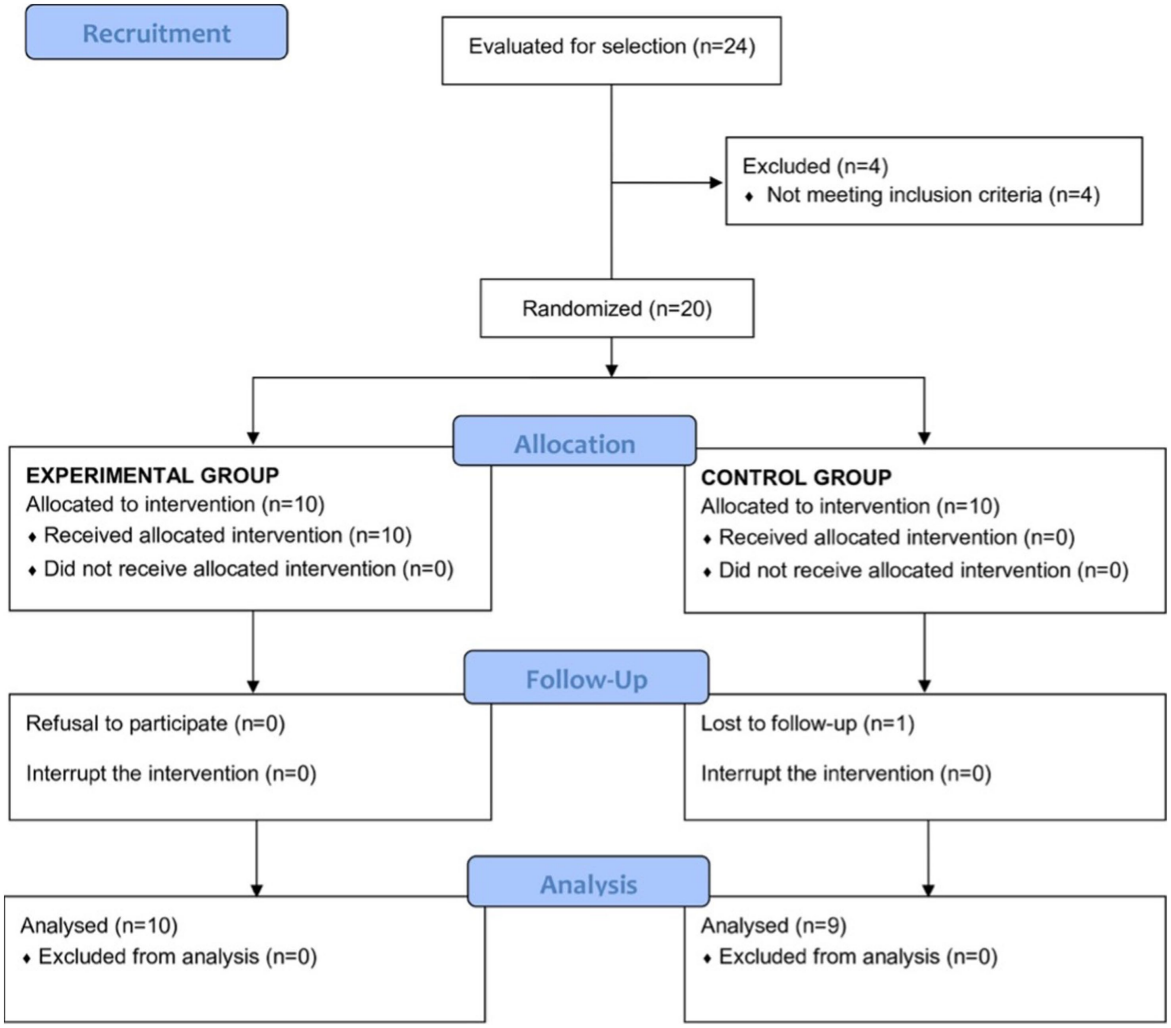
Flow chart of the study population.

A total of 24 voluntary men were eligible, but 4 of them did not meet the inclusion criteria and were excluded. Of the remaining 20 subjects, 10 of each were randomly assigned to the experimental and placebo groups, respectively. However, 1 subject assigned to placebo did not attend the final visit and was lost to follow-up. Therefore, the final study population included 19 participants (10 in the experimental group and 9 in the placebo group), with a mean age of 22.5 ± 3.3 years (experimental group 25.5 ± 2.2 years, placebo group 23.7 ± 4.0 years). The flow chart of the study population is shown in Figure 1.

### 3.4. Serum levels of β-alanine

**Figure 2:**
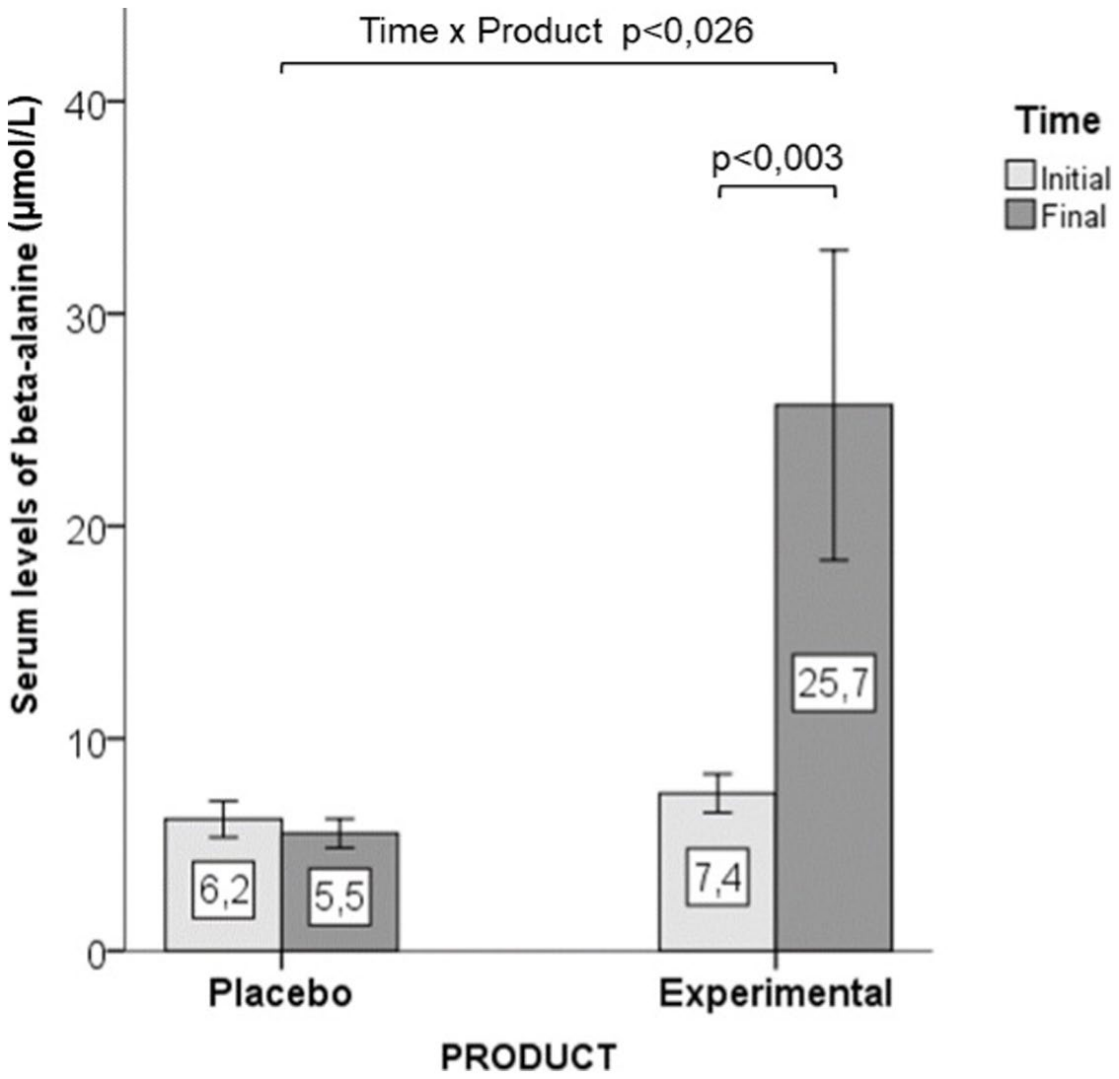
Differences in serum levels of β-alanine between the study groups, with statistically significant within-group (*p* = 0.003) and between-group (*p* = 0.026) differences in favor of supplementation with β-alanine (data as mean ± standard deviation).

Serum levels of β-alanine showed a significant increase in the experimental group, whereas there were not significant differences in the placebo group (Figure 2). Significant time x group interactions (*p* > 0.026) were observed for serum levels.

### 3.5. Changes in laboratory data

Results of laboratory analyses are shown in Table 2.

**Table 2:** Changes of anthropometric variables.

		V1 Baseline	V2 Final	Baseline differences *p* value	Between-group differences Time x Product *p* value
Weight, kg	Control	68.5 ± 2.0	68.2 ± 1.9	0.532	0.718
	Experimental	74.9 ± 2.1	74.4 ± 2.0	0.282	
BMI, kg/m^2^	Control	22.0 ± 1.9	21.9 ± 1.7	0.527	0.594
	Experimental	23.1 ± 1.6	23.0 ± 1.2	0.191	
Fat mass, kg	Control	9.4 ± 3.4	9.3 ± 2.7	0.827	0.854
	Experimental	10.6 ± 2.7	10.4 ± 2.4	0.646	
Lean mass, kg	Control	59.1 ± 1.6	58.9 ± 1.6	0.541	0.769
	Experimental	64.3 ± 1.7	64.0 ± 1.6	0.330	
Muscle mass, kg	Control	56.1 ± 1.5	55.9 ± 1.5	0.568	0.763
	Experimental	61.1 ± 1.6	60.8 ± 1.6	0.343	

BMI, body mass index. Data as mean and standard deviation (SD).

Results of serum hemoglobin levels and white blood cell and platelet counts showed similar values at baseline and at the end of study in the experimental and the placebo groups, without significant between-group differences (time x group interactions). As shown in Table 2, in the β-alanine group, there were statistically significant increases in triglycerides, LDL-cholesterol, and urea nitrogen at the end of the study as compared with baseline. Urea nitrogen also increased significantly in the placebo group. No differences were found between the groups in any of the laboratory parameters (time x group interactions) since increases in these variables were also observed in the control group, although not statistically significant except for urea nitrogen. Deviations from the range of normal values of laboratory parameters are shown in Table 3. Minor abnormalities in laboratory parameters, some of which were already present at baseline, were observed. Most abnormalities were small increases in cholesterol, triglycerides, LDL-cholesterol, aminotransferases, blood urea nitrogen, and uric acid. These abnormalities were observed in both groups.

**Table 3:** Changes of hemogram, lipid profile and liver and renal function tests.

Hematological values		Visit 1	Visit 2	Baseline differences *p* value	Between-group differences Time x Product *p* value
Hemoglobin, g/dL	Control	15.6 ± 0.3	15.4 ± 0.3	0.665	0.828
	Experimental	15.7 ± 0.3	15.6 ± 0.4	0.479	
Leukocytes, x1000/mm^3^	Control	6.2 ± 1.3	5.8 ± 1.3	0.226	0.618
	Experimental	6.5 ± 1.3	6.3 ± 1.3	0.630	
Lymphocytes, x1000/mm^3^	Control	39.8 ± 4.8	36.4 ± 6.2	0.126	0.167
	Experimental	34.9 ± 9.7	36.0 ± 0.4	0.648	
Neutrophils, x1000/mm^3^	Control	50.0 ± 4.8	53.0 ± 6.8	0.155	0.643
	Experimental	54.3 ± 9.3	55.9 ± 9.8	0.457	
Platelets, x1000/mm^3^	Control	244.9 ± 52.7	238.2 ± 43.7	0.420	0.269
	Experimental	218.1 ± 27.3	224.9 ± 29.5	0.439	
Lipid profile					
Cholesterol, mg/dL	Control	158.9 ± 36.9	168.2 ± 35.7	0.077	0.714
	Experimental	161.9 ± 38.3	168.5 ± 41.3	0.220	
Triglycerides, mg/dL^*^	Control	64.4 ± 28.3	80.8 ± 28.9	0.025	0.843
	Experimental	76.5 ± 22.1	90.9 ± 35.6	0.055	
HDL-cholesterol, mg/dL	Control	57.9 ± 8.8	54.2 ± 9.2	0.160	0.843
	Experimental	52.7 ± 9.9	49.7 ± 9.7	0.279	
LDL-cholesterol, mg/dL^*^	Control	88,1 ± 35.4	97.9 ± 32.5	0.043	0.649
	Experimental	93.8 ± 30.5	100.7 ± 32.2	0.171	
Liver function					
ALT, IU/L	Control	28.4 ± 11.1	24.2 ± 10.5	0.272	0.358
	Experimental	21.2 ± 6.9	19.0 ± 7.6	0.096	
AST, IU/L	Control	24.7 ± 6.5	23.4 ± 8.1	0.634	0.594
	Experimental	26.7 ± 8.0	22.0 ± 0.0	0.191	
GGT, IU/L	Control	20.4 ± 7.7	20.0 ± 4.6	0.731	0.555
	Experimental	15.3 ± 3.6	16.0 ± 3.8	0.624	
Renal function					
Creatinine, mg/dL	Control	1.0 ± 0.1	1.0 ± 0.1	0.533	0.493
	Experimental	0.9 ± 0.1	0.9 ± 0.1	0.722	
Urea nitrogen, mg/dL^*^	Control	30.7 ± 8.4	37.9 ± 4.8	0.001	0.265
	Experimental	31.4 ± 8.1	35.5 ± 9.8	0.046	
Uric acid, mg/dL	Control	5.4 ± 1.4	4.9 ± 1.0	0.090	0.101
	Experimental	5.5 ± 1.1	5.1 ± 0.6	0.223	

HDL, high-density lipoprotein; LDL, low-density lipoprotein; ALT, alanine aminotransferase; AST, aspartate aminotransferase; GGT, gamma– glutamyl transpeptidase; * normal values: triglycerides 150 mg/dL, LDL-cholesterol 130 mg/dL, urea nitrogen 50 mg/dL.

### 3.6. Paresthesia

**Figure 3:**
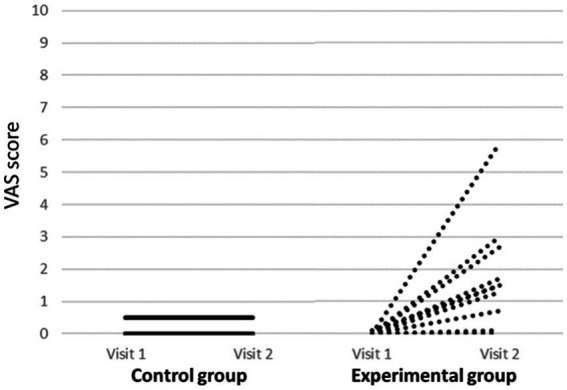
VAS score of each subject belonging to both groups before and after consumption of the product (visit 1 and 2).

There were two participants, one in the experimental group and one in the placebo group that reported having paresthesia at visit 1. In both cases, subjective symptoms were described as tickling. At the end of the study (visit 2), there were 10 participants with paresthesia, 1 in the placebo group (the same subject that reported this symptom at visit 1) and 9 in the experimental group, including the same subject that had paresthesia at visit 1, but with an increase in the intensity of symptoms. Therefore, there were 8 participants who reported “*de novo*” paresthesia after ingestion of the active product. Of the 9 subjects with paresthesia in the experimental group, only one exceeded the VAS score of 3/10 (5.9) (Figure 3). A significant increase in the VAS score was observed in the experimental group. There were statistically significant within-group and time x group interactions in the VAS score of paresthesia (Table 4). Subjective symptoms in the experimental group at the end of the study were described as tickling in 8 cases (88.9%) and as needle prick in 1 (11.1%).

**Table 4:** Number of subjects who presented abnormal values of laboratory parameters and the values of those abnormal parameters.

Laboratory parameter (normal value)		Visit 1	Visit 2
		*N* (anormals values)	*N* (anormals values)
Lymphocytes (25–50%)	Control	0	0
	Experimental	1 (17.5)	2 (23.4; 23.2)
Cholesterol (<200 mg/dL)	Control	1 (210)	1 (243)
	Experimental	2 (207.6; 214)	3 (213.6; 223.6; 227.9)
Triglycerides (<150 mg/dL)	Control	0	1 (169)
	Experimental	0	0
LDL cholesterol (<130 mg/dL)	Control	1 (181.9)	2 (175.4; 131.9)
	Experimental	2 (139.5; 134.3)	3 (130.3; 134.4; 153.7)
ALT (<40 IU/L)	Control	1 (56)	1 (51.3)
	Experimental	0	0
AST (<38 IU/L)	Control	0	1 (43)
	Experimental	1 (38.7)	1 (43.5)
Urea nitrogen (<50 mg/dL)	Control	0	0
	Experimental	0	1 (52)
Uric acid (<7.7 mg/dL)	Control	1 (7.84)	0
	Experimental	0	0

N, number of subjects; LDL, low-density lipoprotein; ALT, alanine aminotransferase; AST, aspartate aminotransferase.

## 4. Discussion

This study shows that supplementation with 15 g per day of a sustained release formulation of β-alanine for 30 days in healthy recreational trained men caused significant increases in the serum levels of triglycerides, LDL cholesterol and urea nitrogen at the end of the study in the experimental group, although without significant differences with the control group; an increase in the number of subjects presenting values of blood variables above the normal range was also observed, but this evolution was the same in both groups. The administration of the sustained-release formulation was associated with minor variations shown in a few patients that consisted of a small increase in the upper limit of normal of aminotransferases, cholesterol, triglycerides, HDL– and LDL– cholesterol, urea nitrogen, and uric acid. Some of these minor alterations were already present at baseline, and in general, more placebo-treated subjects showed alterations of laboratory biomarkers at the end of study than those administered β-alanine supplement. In a study of 14,010 samples from 3,588 elite athletes and 32 sport modalities, in comparison with reference population data, continues exercise affected basal levels of hematological and biochemical parameters with changes in the percentage of eosinophils, creatine kinase (CK), urea, creatinine, aminotransferases (AST, ALT), total bilirubin, lactate dehydrogenase, and potassium (18). These alterations could be more related to exercise than to the ingestion of the experimental product.

On the other hand, an increase in the number of subjects presenting paresthesia (VAS scores below 3/10 in the large majority) and in the VAS score was observed in the experimental group after ingestion of the product.

Two aspects of the study should be commented on. Firstly, the high daily dose of β-alanine of 15 g for a total intake of 450 g during the study period, and secondly, the rate of paresthesia in subjects treated with the active product as compared with placebo. In relation to the dose of β-alanine, in a previous study of our group, 20 g of the same sustained-released formulation was administered during one week (7 days) as a short-term strategy to maintain high-intensity cycling performance (15). In that study, β-alanine supplementation was scheduled in 4 daily intakes and no side effects or paresthesia were reported. However, a pharmacokinetic study of a single oral dose of 8 g of the same sustained-release powder blend formulation produced the expected paresthesia but no other side effects (14). It has been suggested that peak plasma concentration may not be an accurate predictor of paresthesia intensity and that other inter-individual characteristics (e.g., ethnicity) may affect location, timing, and intensity of paresthesia (17). However, the maximum dose that would not trigger paresthesia has still not been identified.

The high daily dose of 15 g of β-alanine was administered in 3 intakes, allowing the use of larger daily amounts and adherence to shorter supplementation protocols. On the other hand, amino acids in sports nutrition supplements are more convenient in powder form, thus providing adequate doses of the active product in single doses, with the advantage of avoiding ingestion of several tablets (14). It has been suggested that the use of sustained-released formulations is more convenient for β-alanine supplementation because higher doses may increase muscle carnosine content in a reduced time, reducing also the symptoms of paresthesia. In a randomized placebo-controlled study in which two protocols of 6 g and 12 g per day of a sustained-released formulation of β-alanine given for 4 and 2 weeks, respectively, the 12 g daily protocol accelerated the increase in carnosine content in skeletal muscle while attenuating paresthesia (19). In a study of 39 recreationally active men and women assigned to three treatment groups of β-alanine: a traditional rapid-release formulation, a sustained-release formulation, and placebo, the change in muscle carnosine content in participants consuming the sustained-release formulation was significantly different from those consuming placebo, and symptoms of paresthesia were significantly more frequent in the rapid-release formulation (20).

In our study, there were two subjects, one in each study group who reported symptoms of paresthesia at the baseline visit. One of these participants assigned to the placebo group continued with the same tickling sensation of the same intensity at the end of study, whereas the other participant assigned to β-alanine supplementation reported an increase in the intensity of paresthesia at the final visit. Although paresthesia was significantly more frequent in the experimental group, the presence of this symptom did not affect compliance with the study product, and was not associated with withdrawal from the study. Interestingly, in a systematic review and meta-analysis of 101 human intervention studies with 1,295 subjects consuming active β-alanine supplement and 973 placebo, the analysis of the incidence of paresthesia was conducted with data of 22 “high quality” studies only (285 and 219 subjects assigned to the β-alanine and placebo groups, respectively), showing an incidence of 18.6% in the active treatment group and 5.7% in the placebo group (21). Meta-analysis showed a significant increase likelihood of paresthesia with active supplementation (odds ratio 8.9, 95% credible confidence interval 2.2–32.6), but there was wide variation in the occurrence and severity of paresthesia (21). However, it was concluded that paresthesia was the only reported side effect, and no evidence exists to indicate that this phenomenon has any adverse consequences (21). However, factors influencing the appearance of paresthesia are difficult to determine given important differences in the design of the studies, doses administered, monitoring of compliance, and how side effects are reported.

Some limitations of the study were that it did not evaluate the effect of supplementation on muscle carnosine (22), nor the potential impact of subjective symptoms of paresthesia on activities of daily living (23), and that the study was conducted only in male subjects. The second limitation was that in our research, we did not use a validated questionnaire, such as the carried out by Décombaz et al. (24), to measure paresthesias; instead, we employed an EVA (Visual Analog Scale) for this purpose. The reason for not utilizing the Decombaz scale was that they are validated to assess paresthesias immediately after product consumption. However, in our study, athletes consumed the product over several days, and we requested retrospective information about the sensations they had experienced during that period of consumption. Consequently, the use of Decombaz’s questionnaire was not appropriate, given the different conditions of use. Nevertheless, we partially incorporated some elements of that questionnaire: Q1: EVA; Q3: the Qualitative Light Symptoms Inventory (QLSI) to qualify the nature of the sensations using six descriptive attributes; Q4: The Body Sensitive Surface Score (SSS) to highlight spatial characteristics and identify the body areas most affected by symptoms. Another limitation that must be acknowledged is the small sample size and the inclusion of only men. In addition, only participants of Caucasian ethnicity were included. Due to the characteristics of the sample, the authors acknowledge that these results are only reproducible for recreational trained men.

## 5. Conclusion

In this study, daily intake of 15 g of a sustained-release formulation of β-alanine during 30 days caused some increases in triglycerides, LDL-cholesterol and urea nitrogen, although there were no differences with the control group. The appearance of paresthesia, described above all as tickling, was the majority but presented VAS score less than 3/10 in almost all subjects. Further studies are required to investigate the changes in blood parameters produced by the intake of high doses of beta alanine.

## Data availability statement

The raw data supporting the conclusions of this article will be made available by the authors, without undue reservation.

## Ethics statement

The studies involving humans were approved by Universidad Católica de Murcia. The studies were conducted in accordance with the local legislation and institutional requirements. The human samples used in this study were acquired from primarily isolated as part of your previous study for which ethical approval was obtained. Written informed consent for participation was not required from the participants or the participants' legal guardians/next of kin in accordance with the national legislation and institutional requirements. Ethical approval was not required for the study involving animals in accordance with the local legislation and institutional requirements because our study did not use these variables.

## Author contributions

FL-R and VÁ-G conceived and designed the study. SP-P, DR-C, and VÁ-G assisted with recruitment of participants. FL-R conducted the statistical analysis and interpretation of data, with input from VÁ-G, LA-C, SP, and DR-C. MD-S drafted the manuscript with input from AM-H, AL-R, FL-R, SP-P, and VÁ-G. All authors contributed to the article and approved the submitted version.

## Conflict of interest

The authors declare that the research was conducted in the absence of any commercial or financial relationships that could be construed as a potential conflict of interest.

## Publisher’s note

All claims expressed in this article are solely those of the authors and do not necessarily represent those of their affiliated organizations, or those of the publisher, the editors and the reviewers. Any product that may be evaluated in this article, or claim that may be made by its manufacturer, is not guaranteed or endorsed by the publisher.
